# The Effects of Arts, Trades, and Professions, and of Civic States and Habits of Living, on Health and Longevity, with Suggestions for the Removal of Many of the Agents Which Produce Disease, and Shorten the Duration of Life

**Published:** 1843-04

**Authors:** 


### BRITISH AND FOREIGN MEDICAL REVIEW,

FOR APRIL, 1843.

### PART FIRST.

### Analytical and Critical Reviews.

### Art. I.

1. *The Effects of Arts, Trades, and Professions, and of Civic States and Habits of Living, on Health and Longevity, with suggestions for the removal of many of the agents which produce disease, and shorten the duration of life.* By C. Turner Thackrah, Esq.—*London*, 1832. 8vo, pp. 233.

2. *Report from the Committee on the Bill to regulate the Labour of Children in the Mills and Factories of the United Kingdom; with the Minutes of Evidence, Appendix, and Index. Ordered by the House of Commons to be printed*, 1832.

3. *The Philosophy of Manufactures: or an Exposition of the Scientific, Moral, and Commercial Economy of Great Britain.* By Andrew Ure, M.D. F.R.S.—*London*, 1835. 8vo, pp. 480.

4. *Report from the Select Committee on the Health of Towns; together with the Minutes of Evidence taken before them, and an Appendix and Index. Ordered by the House of Commons to be printed*, 1840.

5. *Tableau de l'Etat Physique et Moral des Ouvriers employés dans les Manufactures de Coton, de Laine, et de Soie. Ouvrage entrepris par ordre de l'Académie des Sciences Morales et Politiques.* Par M. le Dr. Villermé.—*Paris*, 1840. Deux Vol. 8vo.

*Sketch of the Physical and Moral Condition of the Workpeople employed in the Manufactures of Cotton, Wool, and Silk. A work undertaken by order of the Academy of Moral and Political Sciences.* By Dr. Villermé.—*Paris*, 1840. Two Vols. 8vo, pp. 458, 451.

6. *Third Annual Report of the Registrar-General of Births, Deaths, and Marriages in England, with Appendices. Presented to both Houses of Parliament by Command of Her Majesty.*—*London*, 1841.

7. *Notes of a Tour in the Manufacturing Districts of Lancashire; in a Series of Letters to His Grace the Archbishop of Dublin.* By W. Cooke Taylor, LL.D. &c. &c.—*London*, 1842. 12mo, pp. 299.

8. *Report to Her Majesty's principal Secretary of State for the Home Department, from the Poor Law Commissioners, on an Inquiry into the Sanatory Condition of the Labouring Population of Great Britain, with Appendices. Presented to both Houses of Parliament by Command of Her Majesty.* Drawn up by Edwin Chadwick, Esq.—*London*, 1842. 8vo, pp. 457.

We do not believe that we incur any very serious risk of being pronounced disciples of Jean Jacques Rousseau, if we concede that a high degree of civilization is not unattended by some prejudicial circumstances. These, however, seem to constitute not so much the necessary results of human advancement, as its accidental concomitants. It were paradoxical, truly, to maintain that an extension and enlargement of human capability should operate inevitably to the disadvantage of the race. And, indeed, we feel convinced that all the evils following in the train of advancing civilization are to be regarded, not as something inseparable from and inherent in such a state of things, but as the temporary result of that human imperfection which allows man to become enlightened, not in all things at once, but only progressively. The immense development of mechanical skill within the last half century, the great consequent extension of manufactures, and increase in the number, size, and density of our modern towns, furnish an excellent illustration of that puzzling combination of good and evil which leads some minds absolutely to condemn all human improvement in this direction, and to maintain the paradox that the condition of man is deteriorated by the progressive development of his natural powers. A sound wisdom, however, would apply itself to discover by careful analysis the real source of the attendant evil, and would see if its rectification could not be accomplished, without the necessity of acting upon so contradictory a doctrine as would seem to be involved in the position just referred to. The extent to which the evil in question bears upon the sanatory condition of the population forms certainly no unimportant part of any investigation undertaken with this object; and, accordingly, as falling within our peculiar department, we propose, in the following pages, to examine the facts which, in the present state of our knowledge, seem calculated to elucidate the question as to the effect of manufacturing industry upon health and longevity, and to determine, so far as may be within our power, what are the actual conclusions to which the evidence leads.

Like every other subject identified or intimately mixed up with human interests and feelings, this one has proved itself the fertile occasion of very contradictory statements and opinions. One class of inquirers, guided more, probably, by overwrought sentiments of humanity than by unbiassed judgment, have looked upon the discovery of the steam-engine and its application to the increase of our manufactures as little less than an unmitigated injury done to the bulk of society, accumulating the fruits of labour within the possession of a comparatively small number of capitalists, to the utter destruction of all health and comfort on the part of the actual producers of wealth; whilst, an opposite class, regarding the whole matter from another point of view, and more under the influence of a cool, calculating, and selfish political economy, have maintained that the extension of arts and manufactures in modern times has been an unalloyed benefit to the world, and one carrying with it the least possible amount of disadvantage to the masses engaged therein, compatible with anything that is human. Others again, estimating the subject less controlled by feeling, and more in the spirit of accurate and unprejudiced inquiry, have come to intermediate conclusions, believing that more or less of evil has resulted to great numbers, coincidently with very great advantages to mankind as a whole. Much has been said and written, and adduced in evidence, intended to support every view of the case; and, in a subject of so extensive and multifarious and complex a character, any attempt to sift even the sanatory part of the question within the compass of a few brief pages, so as, even approximatively, to make out the positive facts and conclusions developed in the investigation, must prove, doubtless, both imperfect and unsatisfactory; but, nevertheless, it is one of so practically important a character, in its relation to the well-being of society, that, in spite of its difficulty, we shall enter upon the task, cursorily reviewing the more important matter contained in the publications now before us, in order to see what is the precise information they afford, and how far the inferences deduced seem justified by the premises.

The late Mr. Thackrah, of Leeds, was one of the earliest English writers, systematically to direct the attention of our profession to the influence of employment upon health; and his work cannot fail to be read by all with considerable advantage. It furnishes sure signs of having been prepared with great care and of having been preceded by very attentive observation. But the absence of figures, in the elucidation of the more important departments of the inquiry, diminishes greatly the value of the inferences and opinions. However, there are comprised within the work many very sensible and judicious remarks, and we believe that in general they will be found to be tolerably correct. Mr. Thackrah's experience was gained chiefly in the districts of the woollen manufacture; and, as our present object is mainly to examine the influence upon health of manufacturing industry, we shall here present a brief digest of Mr. Thackrah's statements, occurring in various parts of the volume, relative to the condition of parties engaged in the woollen department.

We are told that, although many kinds of manufacturing occupation lead to the inhalation of dusty particles, the *sorters of wool*, who are occasionally annoyed with dust from the lime, which in some kinds of wool is used for separating the fleece from the skin, do not suffer on this account; and, what is yet more remarkable, that the *scourers*, who are all day in a wet room, exposed to steam and currents of cold air, are sensible of no ill effect; although, in washing the wool, the workpeople have their arms immersed up to the elbows in warm soap and water, and next carry the article into a room about the temperature of 80° Fahr. to dry, and immediately afterwards, often in a state of perspiration, and always without full clothing, turn out into the open air to fetch more wool. Yet, it is stated that, from these great and frequent transitions, they are not more subject than others to rheumatism, catarrh, or pulmonary inflammation. The *woolcombers* work in apartments heated to about 80° by charcoal fires: the wool, after washing, yields a light dust, which annoys the air-tubes, and obliges some persons to leave the employ, but yet the men whom Mr. Thackrah found engaged in the occupation, appeared quite healthy; and it was said that out of a hundred individuals, only two or three were absent from illness. Three or four winters before the period of the author's investigations, typhoid fever had prevailed amongst the woolcombers; but this was attributed to the then low rate of wages, and consequent defect of nourishment. The great heat of their working apartments, Mr. Thackrah did not regard as tending to shorten life, many men from sixty to seventy years of age having been noticed among the workpeople. The sum, indeed, of Mr. Thackrah's statements, so far as they rest upon his own observation, would suggest that the woollen manufacture does not exert any special or undue influence in the production of disease or shortening of life; but, that the individuals whom it occupies are generally in good or in bad health, according to the rate of wages, which, when very low, bring into operations other and well-known causes of disease. On the whole, he considers that the woollen cloth manufacture appears to be less injurious to health than any other, in which so great a number of operatives are collected together. In discussing the influence upon health and life of the cotton manufacture, Mr. Thackrah draws a far less favorable picture. However, as in this department his opportunities of arriving at a correct judgment were comparatively but limited, we shall avoid any examination of his statements in this particular, as some of the other publications before us furnish better materials for basing conclusions upon.

It will be within the knowledge of most of our readers that, at various times within the present century, the effect upon health and life of the cotton manufacture has attracted in this country a more than ordinary share of attention; attributable, in great part no doubt, to its recent most extraordinary rate of increase, and to the revolution it has wrought in modern times in the general condition of those parts of the country wherein it is extensively prevalent. It was found that immense aggregations of individuals had, within a very few years, taken place in districts that had hitherto been almost rural; that the means of subsistence afforded to these dense masses of human beings flowed mainly from the existence of the factory system—a system, it was said, which led to the crowding together of all ages and sexes in heated apartments, leaving the individuals but little opportunity either for rest, fresh air, or healthful recreation. This system was considered to operate with especial disaster to the young, sowing the seeds, where it did not speedily destroy, of future disease, in particular of scrofulous deformity and tubercular phthisis. These notions having powerfully excited the sympathy of many benevolent individuals, both within and without the legislature, this matter has at several times been made the subject of investigation both by parliamentary committees and special commissions; and a vast amount of information, bearing upon the whole question, has in consequence been obtained. We have at present before us, in the shape of official and parliamentary reports, the substance of what has been obtained in the progress of this inquiry. Some of the more important matter, in relation to our present object, we shall proceed to lay before the reader.

Before the parliamentary committee, obtained about ten years ago by the late Mr. Sadler, a great number of medical men and others were examined, with a view to ascertain the effects produced upon the human frame by the operation of the factory system. The medical witnesses almost unanimously condemned and denounced it, as the abundant source of pallid looks, stunted growth, personal deformity, and chronic disease of various kinds. One of the first medical witnesses examined, and one having actual experience of the results of the factory system, was Dr. Thomas Young, of Bolton, in Lancashire; this gentlemen, whose evidence will be found at page 518 in the published report, was asked:

"Q. 'What are your opinions as to the medical effects produced by the hours of labour, deduced as well from the principles of your profession as from your actual observation?'

"A. 'The first effects appear to be upon the digestion; the appetite suffers, the digestion is impaired, and consequent emaciation and debility are induced. Scrofulous diseases are common; I am not aware that this disease would be produced in a sound child born of healthy parents, but if a predisposition to scrofula existed in the constitution, the disease (which might otherwise perhaps have remained dormant in the system) is likely to be called into action.'

"Q. 'Have you observed whether pulmonary complaints are the frequent result of such labour?'

"A. 'They are; for example, consumption and asthma; the latter, however, I have more frequently observed in adults than in children.'

"Q. 'But so as clearly to be traceable, in your judgment, to the particular nature of the employment to which reference is made?'

"A. 'Certainly; to the transition from excessive heat to cold, and the inhalation of dust and cotton flue.'"

The same witness, after dwelling at some length upon the tendency of factory labour to induce scrofulous deformity, and disease generally, stated that "he could not doubt that factory-working tended to shorten life, inasmuch as it tended to produce disease." These seem to have been the deliberately-expressed convictions of an intelligent physician residing in the heart of the cotton districts. So that chronic disease, chiefly of a scrofulous and tuberculous character, is here set forth as having been aggravated and extended by the mode in which the cotton manufacture has spread.

Most of the medical witnesses, however, who were examined before Mr. Sadler's committee, were metropolitan practitioners, whose general reputation suggested them to the committee, as suitable parties to give information upon the physiological effects of certain kinds of occupation. The working of the factory system, however, was, for the most part, known to them only through the descriptions which were afforded; and hence the value of their testimony suffers considerable diminution; although it is that which seems most to have influenced both the legislature and public opinion. There was yet one metropolitan witness, Mr. Malyn, whose early days had been spent in the observation of disease in Manchester, the capital of the cotton manufacture, and this gentleman makes several remarkable statements. The following is from his published evidence occurring at p. 526:

"Q. 'Can you say then, from observation and experience, if there is not a great difference in the health and constitution of the operative classes of society, which is induced by the difference of the length and kind of labour which they have to undergo?'

"A. 'As a practitioner, and as an observer of facts, I can affirm that there exists a marked difference between the operatives of the metropolis and those whose condition you are now investigating. As a physiologist, and as such, reasoning on those facts, I have been led to connect that difference with the nature and duration of their employment.'"

In the progress of the examination, Mr. Malyn is asked if scrofula be not peculiarly prevalent in Manchester, and he replies that "it is to a great degree, nor have I, in the observations I have made elsewhere, ever seen it so common as it is in that town. Speaking from general recollection, I should say I have not witnessed, since my departure from that place, one tenth of what I observed there, although I have ever made it a rule (when otherwise unoccupied), to continue my researches among the poor." This is a startling statement; but we defer all critical remark, and proceed to the views expressed by other witnesses.

Dr. Blundell, whose evidence is published at p. 540 of the Report, is appealed to by the committee for his opinion as to the effects upon individuals, and especially children, to be looked for from prosecuting the kind of occupation described to him by the examinant as "the labour in question, continued for the length of time described, (thirteen, fourteen, fifteen, and even eighteen or nineteen hours a day,) without sufficient and often without any intervals, even for meals, generally pursued in an erect, or at least a constrained position, and in a foul and polluted atmosphere, frequently heated to a very high temperature, and many times continued far into or during the whole of the night." The effects anticipated by the witness are "dyspeptic symptoms, and all their consequences; nervous diseases in greater or less degree; and as the result of both, stunted growth; languors; lassitude; general debility; and a recourse to sensual stimulants in order to rid the mind of its distressing feelings." The examinant, in the progress of Dr. Blundell's evidence, states that "an official paper has been ordered by and delivered to this committee, which shows the great waste of human life, especially at its earliest stages, in the manufacturing or factory districts, as compared with the mortality at corresponding periods of life in other towns and places." We shall return to this assumed fact; and, happily, our national system of registration furnishes much more precise and accurate data, respecting all such subjects, than were to be found during any of the periods at which these parliamentary investigations took place.

In Dr. Hodgkin's examination, beginning at p. 546, a perpetual appeal is made to his anticipations as to the probable effects of the factory system, as represented to him by the querist; and the questions, in most cases, being proposed in the form denominated "leading," we have results expected, certainly of a most disastrous character. These are, great depression of the general health, diminutive stature, varicose ulceration of the lower extremities, scrofulous deformity, premature puberty in the female, leading to most immoral consequences; and the climax attained by the conclusion, that "where the system prevails extensively, considerable abridgement of human life must be the obvious consequence." Mr. Morgan, of Guy's Hospital, is subjected to an examination somewhat in the same strain, furnishing his answers apparently with equal satisfaction to the committee. One observation, however, made by this gentleman is too remarkable to be passed over without notice, and we shall here quote it, for the purpose of reverting to the same, when, in the succeeding pages, we offer our own remarks in the way of commentary. He says, "the employment of a chimney-sweeper I should consider, *perhaps*, more injurious than *even* these manufactories."

Amongst the medical witnesses furnishing their testimony before this committee, the late Sir Anthony Carlisle occupies a prominent position. Although examined at considerable length, there is little in his evidence differing from or in addition to that afforded by the others whose testimony we have noticed. Some observations, however, made on the occasion by this witness, we shall here adduce, as containing, in our belief, rather questionable matter. In discussing the probable effects of the factory system much in the same strain as the foregoing witnesses, he avers, constructively, that, amongst other consequences, the procreative power diminishes considerably, as, in his opinion, it is one of the first faculties to cease with every diminution of animal health and vigour. He volunteers, also, a somewhat remarkable statement, in illustration and confirmation of the above position: "I would offer to the committee," says he, "a matter in my own experience; the city of London would not maintain its population for fifty years, if it was not refreshed by accessions from the country. I have had the curiosity to see if I could find a person of the fourth generation, by both the father and mother's side in the city of London, and I have never been able to find such a person."

The statement on the part of an examining member of the committee, respecting the unusual rate of mortality in the districts where the cotton manufacture prevails, is repeated in yet stronger terms during the examination of the late Sir W. Blizard, to whom it is said, "Wherever this (the factory) system prevails, it is accompanied by an extraordinary degree of mortality, especially in the earlier periods of life; taking the view you have done of the pernicious effects of labour so long pursued, and under the circumstances explained, you would be prepared for that result, namely, a greatly increased degree of mortality?" To this, the witness replies: "I do not know the fact; but, *à priori*, I should have no doubt of it, not the shadow of one." It is also the opinion of Sir William that, in the case of females, factory labour, as described, must lead to a premature attainment of puberty; and in this view he is corroborated by other medical witnesses, and, as before stated, in very strong terms by Dr. Hodgkin.

It is demanded of Dr. Elliotson, by the committee, what he conceives would be likely to result from such a system of labour; and to this he promptly replies, "scrofulous disease of every description, consumption, and deformities."

We cannot, in these extracts, from the report of Mr. Sadler's committee, omit to notice the very decisive character of Mr. Green's evidence, who, after stating that he cannot pretend to any practical experience in these matters, says, "I may yet be able to contribute some information that my professional duties have enabled me to acquire, in aid of your benevolent purpose of duly limiting the hours of infantile and early labour." Upon this, we have a most able and lucid exposition of the causes that excite and predispose to scrofulous disease, with the order in which its various forms are commonly developed, and the whole wound up with the following:—"And lastly, the lungs become the seat of this destructive disease in the form of that incurable complaint of our climate, pulmonary consumption." All this is placed in direct relation to the factory system by the mode in which the answers succeed the questions, and Mr. Green "fears that this country will have much to answer for, in permitting the growth of that system of employing children in factories, which tends directly to the creation of all those circumstances which inevitably lead to disease."

The published evidence of Dr. J. R. Farre is too remarkable to be passed over without notice; one or two of its more striking passages, we here extract: "In English factories," says he, "everything which is valuable in manhood is sacrificed to an inferior advantage in childhood. You purchase your advantage at the price of infanticide; the profit thus gained is death to the child. Looking to its effects, I should suppose it was a system directly intended to diminish population." To the above strong expressions applied to the factory system, we subjoin the following observations having a more extended relation: "I call the British system a forcing system, which departs from the truth of nature, and from the revealed will of God. I have no hesitation in affirming that there is not a due regard to the preservation of life, either in the British system of education or of labour generally, both as regards the child and the adult; and what I say of the adult, applies still more strongly to the child."

The above quotations furnish the main substance of the testimony afforded by the medical witnesses upon the occasion referred to; and, assuredly, if the conclusions to which the whole points, have any material foundation in fact, it is difficult to say what national advantages ought not to be sacrificed, rather than that a system so destructive to humanity, both morally and physically speaking, should be allowed to go on without the application of some grave check. In reviewing, however, the whole of the circumstances connected with the evidence just adduced, it becomes clear that considerable one-sidedness is mixed up with the mode in which the testimony is obtained. The replies of the medical witnesses are almost invariably to leading questions; and, when they refer to factory labour in express terms, it is to the labour as described to them in the exaggerated phraseology of the examining members of the committee, who, but too often, in their way of conducting the investigation, remind one of the proceeding of the advocate in making out a case, rather than of that of the impartial inquirer after the truth. It is strikingly confirmatory of this view of the case, that, out of eighteen medical witnesses examined almost successively, sixteen were metropolitan, and two only from the manufacturing districts; and that one of these two, Mr. Thackrah, was a practitioner resident, not in the district of the cotton, but of the woollen manufacture. Indeed, in going through this evidence, we discover practically, little more than a series of judicious observations upon hygiène, accompanied with strictures upon the way in which its laws are presumed to be infringed by the proprietors of cotton mills.

We have said that considerable one-sidedness attaches to the character of the medical evidence, and that the severity and hardship of factory labour are described in obviously exaggerated phraseology; this would appear from an examination of some of the more important matter contained in the third work, the title of which we have placed at the commencement of this article. The Philosophy of Manufactures, by Dr. Ure, exhibits the results of great attention and labour in the investigation of the whole subject, and some of the author's statements are both curious and interesting, especially when compared with the direful details at which we have just glanced. Whatever undue bias may pervade the work of Dr. Ure, and we think it far from being free from blemish of this kind, it yet possesses the great value of furnishing its facts and conclusions from observations actually made by the author upon the spot, he having, we believe, with a view to the investigation, resided for a considerable time in the very heart of the cotton manufacture. We will contrast, then, Dr. Ure's account of the nature of factory labour, with that given by the members of Mr. Sadler's committee; and we shall afterwards compare the positive statements, as to the effects of the same upon health and life, with the anticipations which the descriptions of the parliamentary committee led metropolitan physicians and surgeons to form.

Now we ascertain, as the result of Dr. Ure's inquiries, made at a period of great manufacturing prosperity and activity, that, instead of the labour being pursued almost through the entire night, it is but rarely carried beyond twelve hours out of the twenty-four; and, that night-work is scouted by all respectable mill-owners, as being equally unprofitable and demoralizing. We learn, moreover, that so far from the atmosphere being unusually polluted within factory walls, there exists in many cases a system of ventilation of very great superiority, as will appear from the following passage:

"The factory plan is to extract the foul air, in measurable volumes, by mechanical means of the simplest but most unfailing kind, especially by eccentric fans made to revolve with the rapidity of nearly 100 feet per second; and thereby to ensure a constant renewal of the atmosphere in any range of apartments however large or closely pent they may be." (p. 380.)

We quote further from the Philosophy of Manufactures, respecting the temperature of cotton-mills; it is observed,

"In Manchester, whenever the temperature of the external air is genial, no artificial heat is used in the fine spinning mills, which never require a heat above 75° Fahr., as several respectable witnesses prove on oath." (p. 400.)

And with respect to that "crowding together of individuals" in the apartments of cotton-mills, upon which so much stress has been laid, Dr. Ure says,

"It is utterly impossible, from the nature of the machinery. The mules, in their advancing and retreating locomotion, must have five or six times the space to work in that the actual bulk of the mechanism requires. Now, nine tenths of the children are employed tending these open-spaced mules. Any one who has once visited a cotton-spinning room must be aware of the impossibility of unduly crowding human beings in a mule apartment. Nor are any of the other rooms crowded with workers, for this plain reason, that no useful purpose could thence result to the manufacturer." (p. 401.)

We will now present to our readers some of the results relating to the health of factory operatives, to which Dr. Ure was led by his investigations. On regarding the morbific effects attributed by large numbers to the prevalence of the factory system, diseases of the character and class ordinarily comprehended in the term "scrofula" seem to constitute the sum of the alleged consequences; thus, the vicious digestion, the nervous debility, and glandular disease; the stunted growth, osseous deformity, and pulmonary consumption; all these, as forming its special manifestations, were the results anticipated by medical reasoners; let us then see whether the testimony of medical observers deposes, with anything like uniformity, to the justness of such an inference. Dr. Ure introduces into his work a letter addressed to certain commissioners of inquiry by Dr. E. Carbutt, physician to the Manchester Royal Infirmary, which letter contains answers to certain queries that had been proposed to him, bearing directly upon this question; portions of this letter we shall here extract:

"I wish to be permitted to make a few observations upon matters not contained in these queries, more especially as to the gross exaggerations of medical witnesses, particularly those of London, on the subject of the diseases of cotton factories. These gentlemen, hardly any of whom have ever had an opportunity of seeing persons employed in cotton factories, do almost universally attribute to factory labour the production of scrofulous diseases. Now the fact is, that scrofula is almost unknown in cotton factories, although the climate of this town and neighbourhood is particularly cold and humid. In a very extensive examination, which I and some other medical men made a few years ago, we found to our surprise, that the cotton factories, instead of producing scrofula, are, in some sort, a kind of means of cure. The late Mr. Gavin Hamilton, who was for thirty-six years, surgeon to our Infirmary, and who, previously to that, had been surgeon in the Queen's Bays, said in my hearing, after examining a cotton factory, 'Gad! we found the factories to be a specific for the scrofula.' In one factory, examined by Dr. Holme, and Mr. Scott, surgeon to the Carbineers, of 401 persons employed, eight persons only were affected with scrofula, with no case of distortion of the spine or limbs. . . . . This remarkable absence of scrofula I presume, with perfect deference to the medical gentleman who is one of your number, to attribute to the dryness and warmth of the cotton factories, to the lightness of the work, and to the superior food and clothing which the superior wages of the workpeople enable them to obtain.

"In addition to the above facts, I may mention that, during the sixteen years I have had the honour of being physician to the Manchester Royal Infirmary, I have, nearly invariably, at the consultations previously to operation, to which the physicians are summoned as well as the surgeons, been in the habit of putting to the patient the question, 'what trade are you of?' especially when the case was that of a distorted limb or joint. To which question the answer has almost never been 'work in a cotton factory,' but almost constantly, 'a hand-loom weaver,' or 'a hatter,' or some other trade." (p. 376.)

The statements contained in the above communication possess, certainly, very great interest. Coming as they do from a practical physician, resident in the metropolis of the factory district, with the best opportunities for arriving at a knowledge of the actual facts of the case, they cannot but carry with them considerable weight and importance. However, we proceed to notice the further evidence adduced by Dr. Ure, in support of his views regarding the influence of cotton mills on health. The subjoined statement is remarkable, but Dr. Ure does not mention the authority upon which it is made:

"During the prevalence of the cholera at Stockport, it was observed that the mill-workers enjoyed a remarkable immunity from the attack; an immunity due to the warm dry air which surrounded them while at work, and to the comforts of their homes. The cholera patients in that town were almost all females employed in private dwellings." (p. 378.)

The following fact, if literally true, constitutes a very singular circumstance:

"Not one of Messrs. Strutt's work-people at Belper was attacked with cholera, while the neighbouring handicraft people and farmers were falling victims to this pestilence." (p. 378.)

There is contained in the work whose contents we are now examining, a very valuable and interesting communication upon our present subject from Mr. Harrison, the inspecting surgeon, appointed for the mills of Preston and its vicinity, in an official report, made some years ago by that gentleman, within the limits of whose jurisdiction it seems that there were 1656 individuals employed in mills under eighteen years of age, of whom 952 were engaged in spinning-rooms, 468 in carding-rooms, 128 at power-looms, and 108 in winding, skewering, cops, &c. Mr. Harrison reports as under:

"I have made very particular inquiries respecting the health of every child whom I have examined, and I find that the average annual sickness of each child is not more than four days; at least, that not more than four days on an average are lost by each child in a year in consequence of sickness. This includes disorders of every kind, for the most part induced by causes wholly unconnected with factory labour. I have been not a little surprised to find so little sickness which can fairly be attributed to mill-work. I have met with very few children who have suffered from injuries occasioned by machinery: and the protection, especially in new factories, is now so complete that accidents will, I doubt not, speedily become rare. I have not met with a single instance out of the 1656 children whom I have examined, of deformity that is referable to factory labour. It must be admitted, that factory children do not present the same blooming robust appearance, as is witnessed among children who labour in the open air, but I question if they are not more exempt from acute diseases, and do not, on the whole, suffer less sickness than those who are regarded as having more healthy employments. The average age at which the children of this district enter the factories is ten years and two months; and the average age of all the young persons together is fourteen years." (p. 379.)

Before closing with Dr. Ure's work, we shall avail ourselves of certain passages, which it contains, taken from Sir David Barry's Factory Commission Report. The following bears strongly upon our present subject:

"There is one thing I feel convinced of from observation that young persons, especially females, who have begun mill-work at from ten to twelve, independently of their becoming much more expert artists, preserve their health better, and possess sounder feet and legs at twenty-five, than those who have commenced from thirteen to sixteen and upwards." (p. 389.)

Further, in noticing the question relative to the growth and development of persons engaged in factory employment, Sir David gives the following as the result of personal inspection:

"Many of the girls were beautifully formed, who had been from ten years of age to maturity in the mill. I noticed five sisters, from thirteen upwards, all employed in the mill from their childhood, every one of whom might be termed a fine-grown girl; some of them remarkable for symmetry and strength. This day I examined carefully and individually 111 girls of the classes stated, with a view to find, if possible, a case in which the plantar arch, (the hollow of the sole,) had been broken down by continual standing, as is stated in the evidence lately printed, (Mr. Sadler's Committee,) to occur sometimes in factory workers. I found many beautifully-formed feet in those who had worked the longest. In no case did the plantar arch seem to have been in the slightest degree disturbed. Nothing but the evidence of my own senses could have induced me to believe that girls, indeed any human beings, worked as stated from nine years upwards, could yet possess in maturity the apparent extreme of high health and vigour, with finely-proportioned forms. It is quite impossible to give an adequate notion of the quickness and dexterity with which two girls about thirteen years of age, joined their broken ends of threads, shifted the pirns, screwed and unscrewed the flies, &c. To supply the place of such artists by new hands would be utterly impracticable, and difficult in the extreme to find a relay of hands equally expert under present circumstances. There is no sameness of attitude, no standing still; every muscle is in action, and that in quick succession." (p. 391.)

We shall here finish our extracts from the work of Dr. Ure, in which he attempts to show that factory labour is not only not conducive to the production of scrofulous diseases, and to the deterioration of the general health, but actually protective against such calamitous results to those who are engaged in it. We may state, however, that he adduces in further support of his views a variety of testimony in addition to that which we have specifically noticed, and that too from persons whose opportunities must have made them practically acquainted with the subject. And, truly, when all this is placed in juxta-position with the evidence which we have also noticed of a totally opposite character, it furnishes materials for a judgment little complimentary either to medical reasoning or medical observation. On the one hand, we have the deepest sympathies of our nature excited, in commiseration of the unfortunate condition of the victims of manufacturing avarice; and, on the other, we are gladdened to the heart that modern advances in civilization, with their mechanical improvements and increased means of production, have, whilst enriching the capitalist, trebled and quadrupled the health and the happiness, and the bodily comforts of the most helpless of our fellow-creatures! What must we conclude amidst such conflicting testimony? We shall postpone the attempt to determine, whilst we further investigate the data upon which to found a reasonable judgment.

The celebrated French statistician, Dr. Villermé, has, within a recent period, applied himself to the examination of the moral and physical conditions of the workpeople, employed in the manufacture of cotton, of wool, and of silk; and the result he has exhibited in the work whose title is prefixed, undertaken by order of the Parisian Academy of Moral and Political Science. Before examining the contents of Dr. Villermé's volumes in so far as they bear upon our present disquisition, we shall submit to our readers portions of the author's preface, which will explain the way in which the inquiry arose, and, at the same time, furnish a kind of guarantee for the accuracy of his observations, as showing the scrupulous exactness with which he seems to have prosecuted his labours:

"The Academy of Moral and Political Sciences, commissioned M. Benoiston de Chateauneuf and myself to make, in the departments of France, certain researches in political economy and statistical science, the object of which was to *ascertain, as exactly as possible, the physical and moral condition of the working classes*. . . . . . . . . . . . For the purpose of rendering our mission more useful, M. Benoiston de Chateauneuf and myself separated. Whilst my associate traversed the centre of France, and the sea coasts, I visited the departments where the cotton, woollen, and silk manufactures chiefly occupy the workpeople. I shall premise, however, by stating the mode in which I proceeded in my researches. It was necessary for me to examine the effects of a work upon those whom it employs, to interrogate misery without humiliating it, and to observe misconduct without irritating it. This task was difficult. Well! I love to say it, everywhere magistrates, physicians, manufacturers, mere workmen were anxious to second me. By their aid, I have been enabled to see, to hear, and to become acquainted with everything. They have furnished me with information, as if in emulation of one another. Some I got by direct inquiry, some I obtained unawares. And such is the care which I was anxious to apply to this investigation that I have followed the workman from his workshop to his dwelling. I have entered there with him, I have studied him in the bosom of his family; I

have assisted at his meals. I have done more; I had seen him in his toils and in his household, I have wished to see him in his pleasures, to observe him in his parties. There, listening to his conversation, occasionally mingling therein, I have been, unconsciously to himself, the confidant of his joys and his sorrows, of his regrets and his hopes, the witness of his vices and his virtues." (Pref. vi.)

Such is the spirit with which Dr. Villermé set out in the investigation; and, after the protestation of so philosophical a procedure, on the part of our author, a very high interest and importance must attach to any testimony bearing upon the subject, which emanates from such a source. We shall examine, then, the results of Villermé's experience, relating to the effects of manufactures upon health, as exhibited in France; and, as the space devoted to the sanatory part of the inquiry occupies but a small proportion of one of the volumes, we shall endeavour to comprise the substance of what the work sets forth, upon this matter, in a very brief compass.

M. Villermé commences by asserting that he had heard much in the course of his researches, relative to the insalubrity of manufactures, especially of the cotton manufacture. He first notices the charge of insufficient ventilation as a cause of disease, and then proceeds to give the result of his own investigations upon this point. After furnishing an account of the precise measures of the various work-rooms, with the mode in which the air is renewed, and the number of individuals which they inclose when in full operation; he concludes that the great bulk of those employed in the cotton mills have a better supply of air at their work than at their homes, and better also than great numbers of other classes of workpeople. In the cotton manufactures, however, there is, he says, one class of workpeople worse situated in a sanatory point of view than the rest; he alludes to the *batters of cotton*, whose occupation necessitates the inhalation of much dust and *flue;* this class he states to include very small numbers, and to be found only in the mills for fine-spinning, a department of factory labour usually considered to be the least healthy, and respecting the morbific influence of which we shall refer to some important statistical documents in the sequel. M. Villermé states that he discovered an entire accordance of opinion respecting the injurious influence of the dusty inhalation, on the part alike of workpeople, of overlookers, and managers, of the masters themselves, and also of medical men. Indeed, he states that the unhealthful influence of *cotton-batting*, whether done by machinery or by the hand, is so generally admitted, that workpeople engaged therein, relieve one another like soldiers called to mount guard. He says, that, whether it be the dust contained in the raw cotton apart from the cotton flue, or this latter itself, which ruins the health of those engaged in the process, the decay of their health is ever certain, and an established fact; that they complain of dryness in the mouth, in the throat, and are seized sooner or later with a cough gradually increasing in severity. "Nevertheless," he says, "I have met in the batting-rooms some men in good health who told me that they had worked there uninterruptedly for many years." The cough is the first symptom of a slow and formidable disease of the chest, which is always relieved on abandoning the work, and altogether cured at its commencement, if the employment be not resumed. The disease, in the progress of its development, assumes all the characteristics of phthisis, and the medical men resident in the manufacturing districts call it '*cotton-phthisis*,' (phthisie cotonnense,) and some '*cotton-pneumonia*.' M. Villermé states that numbers from this cause die in the hospitals, but that he could not ascertain in what proportion. Formerly, cotton-batting was invariably attended with all these inconveniences, but recent improvements in machinery, the author states, have remedied them in great measure; and the number subject to this hurtful agency has now become exceedingly limited. The carding of cotton which leads to some extent, to an inhalation of a dusty atmosphere, is attended, he observes, with a like insalubrity, though to a much less extent. In all the subsequent processes, however, no inconvenience of this kind results.

Our author considers himself in a condition to refute the accusations of unhealthfulness often made against cotton mills on the score of the employment of oils, and certain other greasy materials, which cause disagreeable odours, and thence are considered to operate prejudicially upon those who are subjected to them.

"But, see these people," he says, "interrogate them, interrogate the medical men and other persons who observe the workpeople, and you will very soon be convinced that these things never inconvenience them; that, for the most part indeed, they do not perceive what is so obvious to the senses of strangers; they would, on the contrary, be much more likely to remark their absence if by any chance they should cease all at once. Singular mistakes have arisen in attributing to such causes, diseases induced by protracted labour, want of rest, insufficiency of food and its bad quality, habits of improvidence, drunkenness, and, to say all in one word, wages below the actual wants of the recipients." (II. 208.)

M. Villermé notices in detail the supposed effects upon the health, of the elevated temperature required in certain factory processes. For carding, he says that a heat of from 59° to 61° Fahr. is all that is required; for spinning, however, a higher temperature is needed, a temperature rising in proportion to the fineness of the threads in course of fabrication, and varying accordingly from 64° to 104° Fahr.; the extreme degree, however, is only needed in comparatively few instances; and we believe it to exceed what is ever required in our own mills. It seems that, according to the inquiries made, the results of the excessive heat could not be uniformly ascertained, as sometimes he was told that no other unpleasantness followed than a sort of erysipelatous inflammation about the bend of the thigh, especially in the case of fat persons, and that this necessitated them occasionally to relinquish their work for a short time only; others stated that many workpeople were in consequence obliged to abandon the fine-spinning work altogether; and again it was said that none but the young could endure the high temperature. M. Villermé was informed also, by many medical men, that an unusual quantity of those so employed were attacked with colds and severe inflammation of the chest, as a consequence of the sudden chilling to which they were exposed. In other instances, however, where the excessive heat is not needed, the work-rooms maintain, especially during winter, a much more gentle and agreeable temperature than the operatives enjoy at home. The influence of the monotony and tediousness of factory labour in the production of pale looks, and general languor of the whole system, is slightly glanced at.

The woollen manufacture, in the estimation of our author, is less prejudicial to health than that of cotton, inasmuch as the special causes of insalubrity have less intensity in the one than the other. Thus the batting of wool occasions much less dust than in the case of cotton, so little indeed that the workpeople scarcely feel any inconvenience. Further, the spinning of wool requires only a moderate temperature; and, with few exceptions, the other processes employed in this manufacture require no insalubrious or even unpleasant degree of heat. Good ventilation is obtained in the woollen apartments equally with those of the cotton manufacture. He sums up his observations upon the sanatory condition of this class of operatives, by stating that neither carders, nor spinners, nor combers, nor winders, &c., appeared more subject than others to particular diseases, from the mere fact of their occupation. Any maladies to which they were unusually liable, seemed to arise rather from circumstances common to all persons engaged in any sedentary pursuit, and passing but little time in the open air.

The manufacture of silk has been stated to be especially unhealthy only in the operations of carding the raw silk, and of drawing the cocoons, (tirage des cocons;) but M. Villermé avers that this is not the case to the extent that has been maintained. He considers that much of the ill health experienced by this class of persons depends upon their bad clothing and want of cleanliness. He mentions that the *drawing* process does not ordinarily continue beyond three months in the year; and hence it cannot have that deleterious influence which it might probably exercise if prolonged throughout the year. The operations of batting and carding the raw silk, as observed by himself at Nimes and Montpellier, did not raise dust to that abundant and prejudicial extent which his reading had led him to expect. Ventilation in the silk-rooms does not seem to be so complete or so well managed, as in those of the cotton or the woollen manufactures; and, on this account, many diseases of the respiratory organs are occasioned by the confined spaces and want of renewal of the air, circumstances which overheat the apartments, and constrain the respiration. Some workpeople thus engaged, however, were observed to be in a state of robust health; but M. Villermé was informed, that this was, in some respects, attributable to the fact of a speedy removal to the hospital following any notable derangement of the health. Altogether, both from his own observation, and from the statements he obtained, he concluded that the early processes (and those only) of the silk manufacture were decidedly unhealthy; but of the precise character of the diseases induced, we are not informed; we infer, however, that the ill effects were witnessed in the production of a bad state of the general health, rather than of special affections.

Our author presents us with a brief section upon the health of the hand-loom weavers, the wretchedness of whose occupation is a matter generally known, and universally admitted; especially in the case of those engaged in the weaving of the cotton fabric. Damp cellar-residences, with small but crowded apartments, miserable ventilation, with imperfect supply of solar light,—a state of things falling to the lot of most persons engaged in this work, cannot be otherwise than hurtful in the extreme to their sanatory condition. Indeed, this class of people may almost be regarded as placed at the zero mark of the social scale; and, as a consequence, it may be expected that they are rendered liable to every possible disease to which an utter neglect and violation of the laws of hygiène subjects both the species and the individual.

In offering certain general considerations arising from the foregoing, M. Villermé observes, that no diseases appertain exclusively to manufacturing pursuits; but there are some, he says, which occur more frequently to the workpeople engaged therein, when the conditions in which they live favour their development. It is thus, he goes on, that in the cotton manufacture, cough, inflammation of the lungs, and pulmonary consumption attack great numbers of those who are occupied in the carding and batting of cotton; and, further, that, according to the indications noted by himself, these afflicting diseases make considerable ravages among the piecers, the sweepers, and the cleansers, a result attributable to the inhalation of dust and cotton-flue. Numerous, however, as are the victims of phthisis and pneumonia, he yet regards their premature decease as an evil less deplorable than the extensive prevalence of scrofulous affections among the body of operatives engaged in manufactures. It is a matter of notoriety, how this scourge, in all its hideous varieties, afflicts the inhabitants of great towns, especially the poor, accumulated in narrow streets, in filthy, dark, and ill-ventilated lodging-houses, places but rarely permeated by the solar ray. But manufactures, even in their actual organization, must not be blamed exclusively for this state of things; the evil is not peculiar to them; it was assuredly not less frequent formerly in proportion to the existence of other insalubrious conditions, when the present manufacturing system had not sprung into existence. M. Villermé says that it is chiefly in a mediate, in an indirect manner, that most of these evils arise. The kind of food, the clothing and lodging, the degree of fatigue, the continuance of the hours of labour, and the moral conduct of individuals, are the main circumstances which influence the health prejudicially or otherwise; and with a beneficial character of these conditions, many of the accidental causes of disease would lose their efficacy.

The above comprises the summary of M. Villermé's views and experience, relative to our present subject; and, having a fair amount of data now before us, we shall offer some comment upon the whole, a task for which, we may be allowed to add, a residence and practice of some years amidst manufactures may, in some measure, have qualified us. And, in the first place, we must express our conviction that, in this country at least, the question now before us has, in the various efforts for its elucidation, never been thoroughly divested of extraneous considerations. When the influence of manufactures upon health and life has, at any time, been made the subject of inquiry, it has rarely been discussed and investigated *by itself;* matters bearing upon their general social effects, and questions of political economy, have been so intermingled therewith, that, almost unavoidably, the spirit of party has obscured, in most cases, the perceptions of the investigator, and confused and complicated the entire subject. In what other way can we account for the wonderfully diverse character of the results variously obtained ? results too, not affecting a hidden or remote department of research, nor yet obtained under circumstances allowing with difficulty the customary aids to determine the precise complexion of the related facts; no, but results that concern the best interests of large masses of the community, whose actual condition, upon which the question arises, is observable by all who are anxious for information upon the subject. Whenever such contradictory evidence as to pure matter of fact is accumulated, it is a sure sign that the question is more or less beset by prejudice, and involved in the spirit of party. Let the testimony reviewed in the foregoing pages, presented to Mr. Sadler's committee, be again considered. Do we not find an undoubted perversion of fact pervading the whole, with a subtle commitment of eminent medical authorities to the side obviously espoused by the leading members of that committee? How, otherwise, can we explain the almost universal consent of medical men of undoubted judgment and information, in condemning the manufacturing industry of this country, not as a system complicated with accidental circumstances prejudicial to a sound condition of the human fabric, which the improvements that ever follow upon an advancing civilization may be expected progressively to remove, but as one necessarily tending to produce disease, to shorten life, and to destroy population? How, otherwise, than by attributing the circumstance to some gross perversion of fact, could the interior of a cotton mill have been at all compared with that of a chimney choked with soot, as was virtually done by Mr. Morgan in the notable remark before quoted, that "perhaps the employment of a sweep was even worse than these manufactories." No wonder that Dr. Cooke Taylor, as stated at p. 22 of his recently-published Tour, should have been led by this parliamentary evidence to believe that "mills were places in which young children were, by some inexplicable process, ground—bones, flesh, and blood together—into yarns and printed calicoes."

We do not ourselves approach this subject in the spirit which we here condemn; we have no conceivable interest in the question, direct or indirect, beyond what is inspired by a regard for justice, and an anxiety to separate the true from the false; and, accordingly, we shall proceed in this discussion, in the spirit, we trust, alike of candour and impartiality.

We have said that medical testimony, in sweeping condemnation of the factory system, was obtained by Mr. Sadler's committee, through a perversion of the facts of the case; let us examine this matter a little more closely. We have before quoted the committee's description of factory labour; and, will it, after all the evidence adduced in the foregoing pages, be regarded as faithful? We are able to confirm Dr. Ure's accuracy, when he states that this labour is not, as maintained, "often continued through the night with scarcely any intervals for recreation or meals." If the statement have had any foundation in fact, it must have been in some exceptional instance, under special circumstances. The operatives, as a rule, are engaged, when in full employment—much too long we admit—from six in the morning until seven or eight in the evening, with half-hours' pauses in the mill for breakfast and tea, and an hour for dinner at home, from twelve to one; and this we believe to be now almost universal throughout the districts of the cotton manufacture. This is a state of things far from satisfactory as to hygiènic conditions, for the young more especially; as it allows, certainly, insufficient periods of repose, in which healthful digestion can take place, and out-door exercise be enjoyed. But surely the presence of this evil to some extent does not justify extravagant exaggeration. The occupation is not, as averred, necessarily pursued in any unusually "constrained position;" that it is for the most part tedious and monotonous is certain, but this supplies no data for its especial condemnation. Let factory labour, in these respects, be compared with many other employments, with those of the tailor, of the dressmaker, &c. &c., and, certainly, it will not suffer by the comparison. But the "air is foul and polluted:" let our extracts from Dr. Ure's work as to the state of things in Lancashire, and those from M. Villermé as to France, testify to the contrary. Cotton mills, usually, are well ventilated. Dr. Cooke Taylor, in the work to which we have just referred, says at p. 23, "I should be very well contented to have as large a proportion of room and air in my own study as a cotton spinner in any of the mills of Lancashire."

We are by no means the apologists of the factory system as it stands; we do not coincide with Dr. Ure in his excessive eulogies of this labour, as contrasted with rural pursuits, and, assuredly, we do not believe it, with some, to be curative of, or protective from, scrofulous disease. We think that partisanship has biassed inquirers at the two extremes of the question. The faults of exaggeration and partiality in the selection of illustrations lie grievously at Dr. Ure's door; and, more recently, Dr. Taylor, we think, has betrayed somewhat too much of the convert's zeal in his praises of cotton mills and cotton lords. Having, from errors in the other direction, been, in his own phraseology, first led to regard the former as places where human "bones, flesh, and blood together were ground into yarn and printed calicoes;" and the latter "as the living representatives of the ogres and giants of our nursery tales;" it is no wonder that, when enjoying the hospitality of such men as Mr. Henry Ashworth, of Turton, near Bolton, and inspecting the admirable economy of the mills and cottages belonging to that gentleman, the ordinary consequences of revulsion of feeling should arise, and that mills and their vicinity should, in the warmth of a kind Irish heart and an excited imagination, become as Eden, and the owners thereof as so many guardian angels. There is, however, in spite of certain drawbacks in Dr. Taylor's work, much valuable matter, exhibiting the folly of the monstrous portraitures of factory labour drawn some years ago, the effects of which upon public opinion are yet far from being extinguished.

Whilst we maintain that many of the conditions of health are as little violated by manufacturing industry as by the immense proportion of other pursuits, we still believe that factory labour is on many accounts injurious; that is to say, when contrasted with the state of the classes raised above that of the operative. How, indeed, can it be otherwise, when regarded as a whole? Individuals thus employed do not spend in the open air more, on an average, than an hour or an hour and a half, in the twenty-four; work is resumed almost the moment the meals are consumed, allowing but little rest for the initiation of a sound digestion; and, in the case of the young, there is nearly an entire absence of that delightful "child's-play," which the purest feelings of our nature reveal to us as so essential to juvenile health, vivacity, and vigour. Causes like these must and do depress, more or less, the vital energy, and induce, certainly, a lower state of the general health than would exist with the presence of an opposite state of things. But, in all this, we have nothing peculiar to the factory system. With the great majority of the working classes in large towns these causes have a general operation.

There are the foundries, where machinery is constructed; there are the joiners' shops, the dye-works, the hat manufactories, the tailors' and milliners' work-rooms; there are the letter-press-printers' apartments, the compact counting-houses; indeed, with few exceptions, the conditions of every kind of labour, in any of our modern cities, present almost the same exceptional points as those of our manufactories, some being more or less favoured in particular respects.

But, it may be demanded, do we believe that no evils occur, specially, to health and life, from the way in which our manufactures are conducted? We frankly confess that the result of all we have read, and all we have seen, has been to convince us, that diseases, peculiarly attributable to manufactures, are not to be found amongst the population dependent upon them. And yet the opposite opinion prevails extensively, and has received countenance and support from the express statements of many who have made this subject a matter of investigation. We cannot help thinking, however, that preconceived notions have biassed the judgment in these cases, when conclusions have had to be drawn from the facts observed. We think that a certain effect has been declared, because, reasoning from analogy, it seemed fairly to be expected. A state of things, calculated to affect a novice in a certain way, does not necessarily produce a similar effect upon the experienced; this wonderfully accommodating principle in the animal economy is too often overlooked; and, hence, it is remarkable how little accordant with the anticipation is the actual condition of many classes of workpeople. Thus M. Villermé informs us, that he had read a great deal of the insalubrious influence of the woollen manufacture; yet his observations furnished no corroboration of the alleged fact. We have all, in our minds, associated the inhalation of minute dusty particles with the development of protracted bronchial irritation; and yet, according to a foregoing extract from Mr. Thackrah's work, the sorters of wool, who are thus annoyed, do not suffer in their health on this account. Exposure to damp and currents of air, with sudden transitions from heat to cold, we ever regard as direct causes of pulmonary inflammation and rheumatic affections; and such would undoubtedly be the result to parties unaccustomed to such exposure; but habit, in such instances, exerts a vast preservative influence. Thus, we are told by Mr. Thackrah that wool-scourers, so situated in an eminent degree, are not more subject than others to such affections. There is no question whatever that this law of accommodation operates only within certain limits; but still there is such a law, and the extent of its influence should be fairly watched and estimated in all such inquiries as the present.

We conceive, for our part, that the great mass of disease constantly witnessed in the districts where manufactures prevail, and so often laid to their account, flows rather from the *great-town* than the *factory-system.* As we said at the commencement of this article, we concede that a high degree of civilization is not unattended with some prejudicial circumstances; and, among the most prominent of these, we regard the rapid springing into existence of our modern towns, which, being the result chiefly of commercial enterprise, arrange themselves for the most part with but little regard to the conditions of health. The attention of the government is now being fast drawn to the consideration of this question; the results of some parliamentary investigations are now before us; let us slightly glance only at some of these evils, and we shall see causes in operation for the production of disease in towns, far more potent than any that originate essentially with manufacturing industry. Thus, in the Report made by a committee of the House of Commons upon this matter, some two or three years ago, we find the following amongst other passages, illustrating the special causes prejudicial to health, in the actual state of our large towns:

"Evidence of undoubted credit, and of the most melancholy description, has been laid before your committee, showing the neglected and imperfect state of the sewerage, paving, and cleansing in many parts of London inhabited chiefly by the working classes; and similar evidence applies with more or less force to many other great towns, the state of which has been investigated, as Dublin, Glasgow, Liverpool, Manchester, Leeds, Bradford," &c. (p. vi.)

The committee dwell at some length upon the dense crowding of cellar and other imperfectly-ventilated residences, as the abundant source of disease in all its varieties, the ill consequences of which are further aggravated by the causes enumerated in the extract we have just given, occasioning damp, the accumulation of filth, and other well-known sources of disease. It is impossible that causes of this kind can be in constant operation, without a terrible deterioration of the health on the part of those who are subjected to such noxious influence; and, we feel well convinced that here will be found the effective agents in the production of disease amongst persons engaged in manufactures. The following passage from the parliamentary Report is of some importance as bearing upon this inquiry:

"In Manchester, the capital as it may be called of the cotton trade, with a population of not less than 240,000, nearly 15,000 of the poorer inhabitants constantly inhabit cellars. Though *the habitations of the working classes are described as better than those of Liverpool*, the want of proper building regulations, and any effectual sewerage and cleansing, as applicable to the localities inhabited by the workmen, is most justly complained of." (p. x.)

Manchester and Liverpool form admirable instances for comparison, in regard to our present purpose; they have a somewhat corresponding amount of population, and the towns occupy very nearly the same superficial extent; the one has been created and lives by the factory system, the other contains, we believe, but one factory, and that of very recent construction; yet the returns of the registrar-general exhibit a higher rate of mortality, and a presumably greater prevalence of disease in Liverpool than in Manchester; and the passage in the above quotation, which we have rendered in italics, indicates a greater abundance of the ills of the great-town system.

In speaking of the condition of factory operatives, we have stated our belief that, on several accounts, their position was unfavorable to health and longevity; but that, in this regard, they differed but little, if at all, from other classes of workpeople who were exposed to the same injurious influences, excepting those supposed to flow especially from the factory system. Mr. Chadwick, the secretary to the Poor Law Commission, in the Sanatory Report, noticed at some length in the present Number of this Journal, has collected certain statistical tables which show that the value of life is greater in the rural than in the town districts; and that, on the average, professional persons and gentry, with their families, attain a higher age than do tradesmen and farmers with their families; and that these latter again have better chances of life than the working classes. Now these facts are most valuable as showing the source of many fallacies that have arisen in the discussion of such questions as our present one; they show that a high mortality may prevail in a particular locality, not because it is the seat of some special department of industry, but because its working population, irrespective of the particular employment, may unduly preponderate. They show that, as a rule, the lower the position of any individual in life, the less favorably situated may he be presumed to be in relation to the conditions of true health; and, upon detailed examination, they show that as the peculiar evils of the "great-town" system abound, the value of life diminishes accordingly; and, assuredly, Mr. Chadwick's figures do not make an unduly unfavorable exhibition of the towns where manufactures prevail, when contrasted with non-manufacturing towns similarly conditioned in all other respects.

The tables in question were obtained from careful analyses of the registration books, by the clerks of the several poor law unions, who at the same time act as superintendant registrars. We shall select a few examples, in illustration of what we have set forth; thus, in the returns of the average age of death amongst the different classes of people in manufacturing Manchester and agricultural Rutlandshire, the figures stand so:

| | *Manchester.* | *Rutlandshire.* |
|---|---|---|
| Professional persons and gentry, and their families | 38 | 52 |
| Tradesmen and their families (in Rutlandshire, farmers and graziers, are included with shopkeepers) | 20 | 41 |
| Mechanics, labourers, and their families | 17 | 38 |

Now, if in the exhibition of the relative mortality of the two districts, no account were taken of the different position in life of the various classes of the population, but the low average of life in Manchester set forth, in comparison with what obtains in Rutlandshire, manufactures in all probability would be referred to as the cause of such a state of things; and, indeed, this was the actual mode of proceeding adopted by the members of Mr. Sadler's committee; certain returns, very imperfect in themselves, were adduced, and contrasted with others not legitimately comparable, because, with the exclusion of the factory system, not similarly related to the other possible causes of disease and early mortality.

The analysed results of our national system of registration have now clearly demonstrated that, in this country, a densely-populated district is less favorable to life than one but thinly inhabited; and the figures just quoted show that the result occurs, in a greater or less degree, in all ranks of life; and thus, in Manchester, where human beings are densely congregated, influences unfavorable to longevity extensively prevail; for we see that the value of life with the most favoured classes is not greater than with the least favoured in Rutlandshire. If it had appeared that, in the higher grades, there was little variation in the average age of death in the two localities, the difference in the pursuits of the workpeople in these places might to some extent have confirmed the idea regarding the specially injurious tendencies of manufactures. But the above facts, with many others of a like character, go to show that the evil appertains to *towns* rather than to *factories*.

Bethnal Green, a compact and thickly-populated part of the metropolis, a district where the existing evils of the great-town-system, so far as the poor are concerned, prevail to a very great extent, exhibits a highly unfavorable picture of human longevity, especially in the instance of those peculiarly subject to the evils in question. The following applies to Bethnal Green: from a population of 62,018,

| | *Average age of Death.* |
|---|---|
| " 101 gentlemen and persons engaged in professions, and their families . . . . . | 45 years. |
| 273 tradesmen and their families . . . . . | 26 |
| 1258 mechanics, servants, and labourers . . . . . | 16." |

From the above it seems that whilst in the first and second divisions the chances of life are greater in Bethnal Green than in Manchester, with the third a lower figure is attained. There are yet no factories in this place, the manufactory being chiefly domestic in the department of silk weaving.

As connected with the question relative to the prevalence of disease and death in manufacturing towns, and as tending to decide whether these evils flow essentially from the factories or from other causes, we will take Mr. Chadwick's figures and prefatory remarks as affecting Liverpool, the most appropriate subject of all for comparison with Manchester, for the reasons before expressed. The following occurs at p. 159 of the Sanatory Report:

"But in Liverpool, (which is a commercial and not a manufacturing town,) where, however, the condition of the dwellings are reported to be the worst, where, according to the report of Dr. Duncan, 40,000 of the population live in cellars, where one in twenty-five of the population are annually attacked with fever, there the mean chances of life appear from the returns to the registrar-general to be still lower than in Manchester, Leeds, or amongst the silk weavers in Bethnal Green. During the year 1840, the deaths, distinguishable in classes, were as follows:

| | *Average age of deceased.* |
|---|---|
| " 137 gentry and professional persons, &c. . . . . | 35 years. |
| 1738 tradesmen and their families . . . . . . | 22 |
| 5597 labourers, mechanics, and servants, &c. . . . | 15." |

Do not facts like the above justify and confirm the opinion which we have expressed that "the great mass of disease constantly witnessed in the districts where manufactures prevail and so often laid to their account, flows rather from the *great-town* than the *factory* system?" Do they not suggest that agencies more powerful than manufactures are at work, whose effects, in the language of Dr. Farre applied to factories, go to "diminish population." Mr. Chadwick has some remarks so applicable just here, that we cannot resist quoting from him at some length:

"One most important result of such investigations would be to disabuse the popular mind of much prejudice against particular branches of industry arising from the belief that causes of ill health really *accidental* and removeable, and sometimes unconnected, are *essentials* to the employment itself. By pointing out the real causes, warning will be given for their avoidance, and indications extended for the application of more certain remedies. Medical men who see only a few patients of the same occupation at distant intervals; who see them in their own dispensaries or in the hospitals, and who have no opportunities of observing such patients under the varied circumstances in which the disease may have been contracted, are left to mere guesses as to its cause. A working person of any of the classes whose condition I have described, presenting himself with the symptoms of a consumption, the medical man has no means of detecting *the* one of many causes by which it may have been occasioned, and the individual patient himself is more likely to mislead than to inform him. Unless his attention were accidentally directed to it, or unless the medical investigator had himself the means of observing the different personal condition of the different sets of persons following the same occupation in town and in country, it is highly probable that the evidence that the disease is not essential to the occupation would escape him. Thus, between different sets of workmen who work at the same descriptions of work during the same hours, and in the same town, but in well or ill ventilated factories a marked difference in the personal condition and general health of the workpeople has been perceived. Great differences are perceptible in the general personal condition of persons working during the same hours in cotton mills in towns, and in cotton mills in rural districts, where they have not only a purer atmosphere, but commonly larger and more commodious places of abode. The factory superintendants generally state that the workers in the country mills are distinguishable at sight by their more healthy appearance, and by the increased proportions amongst them who have florid complexions. Very lately the attention of the Austrian government was called to the labour of persons working in the cotton-factories in the neighbourhood of Vienna. One half, perhaps, of the mills are of the ordinary construction of the cotton mills in England of from thirty to forty years' date, and they work on the average as much as fifteen hours per diem. But it appears that the houses in which the workers live belong to the capitalists who own the mills, many of whom have displayed a desire to ensure, as far as the state of the private residences can ensure, the comfort of those whom they employ, and they have accordingly built for them a superior description of tenements. It is stated that the result of the inquiry conducted by the government physicians was, that the average health enjoyed by the workers in those mills is greater than that of any other class of workpeople in the neighbourhood where the mills are situated, and where the general condition of the population is deemed good; the difference in the general health of the two classes (indicated by the proportions of death—of 1 in 27 of the general population, and 1 in 31 of the manufacturing population,) was ascribed to the difference of the residences." (Sanatory Report, p. 113.)

That factory labour in towns contrasts very unfavorably with agricultural pursuits, we have already maintained in opposition to Dr. Ure; and that, with many other employments, it tends, under present circumstances, to deteriorate the race, we believe to be undoubted; the following from Mr. Chadwick's work is valuable, as illustrating this position:

"In the evidence of recruiting officers, collected under the Factory Commission of Inquiry, it was shown that fewer recruits of the proper strength and stature for military service are obtainable now than heretofore from Manchester. I have been informed that of those labourers now employed in the most important manufactories, whether natives or migrants to that town, the sons who are employed at the same work are generally inferior in stature to their parents. Sir James M'Grigor, the Director-general of the Army Medical Board, stated to me the fact, that 'a corps levied from the agricultural districts in Wales, or the northern counties of England, will last longer than one recruited from the manufacturing towns, from Birmingham, Manchester, or near the metropolis.' Indeed, so great and permanent is the deterioration, that out of 613 men enlisted, almost all of whom came from Birmingham and five other neighbouring towns, only 238 were approved for service." (p. 185.)

Birmingham, however, it will be remembered, is not the seat either of the cotton, wool, or silk manufacture; it is yet one of our great towns. It would be tedious to reiterate the obvious inference.

Before we close the present disquisition, we shall examine somewhat more in detail the specific evils alleged to result from the atmosphere of cotton mills; these being particularly, the premature attainment of puberty on the part of the female, osseous deformity, consumption, and scrofula; we shall offer some remarks upon each of these allegations respectively. With regard to the first, we are satisfied that considerable mistakes exist; we believe that the whole subject is one upon which many erroneous views prevail. It is a notion almost universally prevalent that puberty occurs regularly, at an early or late period of life, according to the high or low temperature of the place of habitation: it is taught for example, systematically, that with the negress, the appearance of the catamenia occurs some years earlier than with the Caucasian, in consequence of the great heat of the climate in the former instance. This generally received statement, however, there is very good reason for believing rests upon no very satisfactory foundation. On the contrary, Mr. Roberton of Manchester, in a paper read before the British Association at its last meeting held in that town, and since published in the Edinburgh Medical and Surgical Journal, brought forward a series of facts well authenticated, and promiscuously gathered, which go far to discredit entirely this commonly received idea. Our space will not permit us to detail the facts in question, nor is it necessary for our present purpose that we should; suffice it is say that, in our opinion, Mr. Roberton has clearly shown that the notion respecting the influence of temperature upon the period of puberty is at least *not proven*; and, further, that it is probable that the idea is a mere conclusion from the analogy of the vegetable world, having no sure basis whatever in direct observation. It is, at any rate, quite certain, that the effect of factories in this respect has been inferred from considerations of this kind. Mr. Roberton, with abundant opportunities, has made this matter one of direct investigation, and it has from him received no confirmation of the anticipations and positive averments of Mr. Sadler's witnesses. We ourselves have again and again proposed the requisite inquiries for obtaining correct information upon this head, in the full expectation of learning that, with factory girls, the age of puberty was much oftener below than above fourteen. We have kept no numerical records of the result of our inquiries, but we are sure that this was, in the great majority of cases, in direct opposition to our anticipation. In a word, we have the fullest conviction, obtained from many opportunities of observation, that this particular charge against factories is entirely unfounded.

On the subject of osseous deformity with the inmates of cotton mills, we have already adduced considerable evidence to discredit the notion that it is so constantly and uniformly met with as the Report of the Factory Committee would have led us to believe. With respect to this matter, some very interesting information was brought before the British Assocation at its Manchester meeting by Mr. Alderman Shuttleworth of that town, whose paper read upon the occasion, is so replete with valuable facts bearing upon the entire subject, that we shall here introduce portions of an abstract of the same, taken from the work of Dr. Taylor before noticed; by doing which, we shall enable the reader to judge, in some measure, respecting the extent of the deleterious influence attaching to the fine spinning mills,—that department of the cotton manufacture commonly regarded as the most prejudicial to health, and most conducive to the development of the special evils of the factory system. The following is extracted from page 258 of the work of Dr. Taylor ;

"The tables which I have to present to the section (the statistical) relate to the nineteen cotton mills in Manchester, which are engaged in spinning fine numbers of yarn. These are the whole of the establishments in this town so employed. As such mills require to be kept at a higher temperature than is necessary in spinning common numbers, it has been generally considered that the health of the workpeople engaged in them was exposed to more injury than in any other kind of factory labour. In consequence of this prevailing opinion, and as the conditions under which the fine spinners are placed are certainly somewhat peculiar, it was thought desirable, when the Factory Commission was appointed in 1833, to collect a body of information which should be confined exclusively to them as a separate and distinct class of spinners. The parties concerned in the inquiry, anxious to have the facts collected under such circumstances as to entitle the statement of them to every degree of confidence, requested me, as a person wholly unconnected with the spinning business, and having no interested feeling in the result of the investigation, to undertake the responsibility of conducting it. I accordingly drew up a series of questions, to be answered personally and individually by each operative spinner, to agents, consisting of professional accountants, and one of our most respectable and intelligent surgeons, who were employed to go through the mills and receive the answers from the workmen. That the answers might be given considerately and after due preparation, every spinner was furnished with a list of the questions a day or two before the agents visited them to receive their replies. The facts and statements, thus most carefully and scrupulously collected, were then arranged by me under the heads exhibited in the tables, and afterwards delivered in evidence before the factory commissioners sitting in Manchester at the time, and their accuracy verified on oath by myself and the agents employed. ........The nineteen mills in question worked sixty-nine hours per week ; they employed 837 spinners who are adults, of whom—

| | | |
|---|---|---|
| " 16 | are under | 21 years of age. |
| 176 | from | 21 to 25 inclusive. |
| 198 | ,, | 26 ,, 30 ,, |
| 155 | ,, | 31 ,, 35 ,, |
| 152 | ,, | 36 ,, 40 ,, |
| 89 | ,, | 41 ,, 45 ,, |
| 33 | ,, | 46 ,, 50 ,, |
| 12 | ,, | 51 ,, 55 ,, |
| 5 | ,, | 56 ,, 60 ,, |
| 1 | ,, | above 60 ,, |

"Of the total number of spinners, 255, or nearly 30½ per cent. were absent from work on account of sickness in the year 1832, an aggregate of 6296½ days, or an average of 24½ days for each of the 255 who were sick, or 7⅛ days for the whole number of spinners employed. Of the 837 spinners, 621 or 74 per cent. reported themselves to enjoy '*good*,' 171, or 24 per cent., to enjoy "*pretty good*," and 45, or about 2 per cent. to have '*indifferent health.*' ...........The married spinners had had 3166 children.........640 of these had worked in cotton mills, and 58 had worked at other employments. Out of the 640 who had worked in mills, 18, or about 2¾ per cent. were dead ; and out of the 58 who had worked at other employments, 4, or nearly 7 per cent. were dead. *The cases of distortion were* 8, *or* 1¼ *per cent.*, and there were 7 cases, or rather more than 1 per cent. of mutilation from machinery."

The above facts, obtained with such precautionary care, and showing as they do certain results relating to the noxious agency of the most noxious class of cotton mills, are of very considerable importance. They furnish positive numerical data respecting matters upon which so much vague declamation had seen hazarded; and they exhibit in a very ridiculous light, the exaggerated statements that had gone abroad, regarding the terrible influence of factories in the destruction of infant life, and the woful distortions of limb.

Among the peculiar evils of the factory system, the production, or premature development of phthisis, seems to have been more generally dwelt upon than any of its other presumed ills. The facts and statements of M. Villermé, noticed in the preceding pages, give an especial prominence to this presumed result of the cotton manufacture, traceable, in his view of the case, mainly to the bronchial irritation caused by the inhalation of dust and cotton flue, and exhibiting itself in a high degree among the batters and carders of cotton, classes of workpeople mainly subjected to this agency. We confess, for our part, that when, in the discussion of any matter of this kind, great stress is placed upon the reasons why a certain consequence must follow, we are generally led to mistrust the accuracy of the fact itself. We always suspect that the observer's attention is excited unduly by instances that seem to realize the expectation, and that what furnishes no corroboration of his anticipations is but little regarded. Medical men, in such cases, too commonly ascribe the results in the language of Mr. Chadwick, "of a cluster of causes to one; and in respect to several classes of workmen, the real cause, the invariable antecedent, is unnoticed." And thus—with minds prepossessed with the notion that tubercular consumption originates essentially in bronchial irritation, and that the atmosphere of cotton mills is exceedingly conducive to the development of this latter—every case of phthisis, occurring among the factory population, is sure to be attributed to the one presumed cause. We ourselves believe that, in the present state of our information this peculiar influence of cotton mills is, to say the least, entirely without proof; and, so far as our information extends, we think that any impartially selected, or promiscuously gathered body of facts would go to show that the idea receives no support whatever, when fairly put to the test. Amongst other communications elucidating this affair of factories, read at the British Association's last meeting, a paper, since published in the Journal of the Statistical Society, was read by Mr. Noble of Manchester; and as this paper illustrates, in some degree, this particular point, we shall here insert certain portions.

After exhibiting figures from the returns of the Registrar-general, showing that Manchester presents no unusual amount of deaths from consumption, as compared with other places, Mr. Noble thus sums up the issue:

"These numerical statements, of unquestionable authenticity, drawn from the national records of the causes of death in various parts of the kingdom, supply certainly no corroboration of the views that have been set forth as to the extraordinary prevalence of consumption in these districts, as compared with other localities differently situated in respect of manufactures. Manchester and Salford seem, on the whole, rather more exempt from the affection than some other places; decidedly more so than Liverpool; but, in comparison with the agricultural districts and the metropolis, they seem more subject to its prevalence. It is a remarkable fact, however, that the metropolis excepted, Manchester has fewer deaths from consumption, in proportion to the whole number of deaths, than any of the other districts above instanced; and, compared with the metropolis in this respect, the ratio is the same."

With a view of ascertaining whether, of the registered deaths from consumption in the township of Manchester, the factory population yielded an undue proportion, an examination had been made of the death books for the years 1838, 1839, and 1840; and the cases registered as deceased from "consumption;" "decline," and "phthisis," had been taken therefrom and recognized as cases of consumption; those below fifteen and above forty years of age at the time of death, having been excluded. Mr. Noble, in explanation of this proceeding thus expresses himself:

"It is reasonable to suppose that, if occupation of any kind operate so as to shorten life through the production of such a disease as consumption, the affection will be developed and terminate fatally within those periods; and, again, I considered that in omitting, for the purposes of this inquiry the instances marked 'consumption' &c. below fifteen and upwards of forty, I should be most likely to embrace the largest amount of real cases, seeing that, in the record of the causes of death, infants of the tenderest age, even those below twelve months, are very frequently registered as having died from consumption; and, on the other hand, persons of very advanced years are similarly registered; there being a high probability that, in both these extremes, a large proportion of such instances have not been real cases, but in the example of children probably some chromic mesenteric affection, and in that of persons past middle life cases most likely of chronic bronchitis with general decay. Not that I feel any confidence of having obtained by the present plan instances only of true consumption, for many deaths from other organic affections, especially of the heart or liver, are in all probability registered, on the imperfect report of those supplying the required information, as from 'decline'; and others, real consumption, are probably given in as 'disease of the chest' or 'weakness;' but, on the whole, I believe that, as regards *numbers*, a very fair accuracy will be gained, the improperly included being balanced by those unduly excluded."

After this explanation of the way in which he was guided in prosecuting the analysis, Mr. Noble goes on to say that,

"The township of Manchester with a population of about 160,000, and with an average of deaths annually of 6000, afforded 1141 registered deaths from consumption in the three years before mentioned; and, as nearly as can be estimated, 174 of these occurred to individuals working in factories, a proportion somewhat more than one seventh of the whole; whilst 590 were of persons registered as of various occupations; and 377 without any stated employment, having for the most part been wives and children not attached to any particular pursuit. Of these 174 inmates of factories, the spinners constituted 45, the winders 49, the piecers 28, the reelers 15, carders and frame tenders each 11; 10 were stated to have wrought in factories without there being any mention of the precise occupation; and the remainder were of doublers, stretchers, batters, &c.; none of them exceeding 5 in number...........Now, when it is considered that the actual *township* of Manchester includes the more central and dense part of our population, I do not think it too much to say that of the inhabitants between fifteen and forty, not very much less than one seventh of the whole are employed in factories; and, if so, no corroboration is afforded of the notion that consumption is disproportionately prevalent amongst the factory population."

Mr. Noble afterwards proceeds to show by further analysis, that the population of Manchester is not at all more liable to *early* invasion of the fatal malady than that in other places, contrary to the general idea entertained upon the subject.

The above facts show satisfactorily to us that, whatever may occur in the manufacturing towns of France, where probably certain other conditions of health may be less favorable than in this country, the factory system does not lead necessarily to the undue prevalence of consumption. In no place do cotton mills abound so extensively as in Manchester, leading to the presumption that if there be anywhere as mentioned by M. Villermé, a "*cotton-phthisis*," it is an accidental and not an essential appendage.

We have a few words to say respecting the extraordinary amount of scrofula in the manufacturing districts, and the unparalleled abundance of cases to be noticed in places like Manchester. Now we can easily understand how Mr. Malyn saw an immeasurably larger number of cases of scrofula when in Manchester, than during his subsequent metropolitan career, as stated in his evidence before parliament. In Manchester, he tells us, that, as physicians' clerk at the infirmary, his business was extensively with the most destitute classes; we presume that his professional pursuits were somewhat of a different character in London, bringing him at any rate into communication less with the lowest social grades; on which account he would not be so likely to encounter scrofulous disease as under his former circumstances. In all investigations of this kind, however, we can attach but little value to the results of partial observation, or to any aggregate of facts which have not been obtained generally and promiscuously.

With a view to illustrate this present matter, we propose to select certain figures from the Registrar-general's report for 1839, now before us; and we shall employ the returns of the deaths from consumption a little differently from what Mr. Noble seems to have done in the paper from which we have just quoted. Some little acquaintance with popular modes of dealing with these matters satisfies us that the cases of death reported to the registrar as *decline* or *consumption*, are representative of general scrofulous disease rather than of phthisis exclusively. A consumption or decline, with the mass of the lower orders, is expressive of most diseases unaccompanied by acute symptoms, and marked by progressive emaciation; and we are sure that, where the scrofulous taint prevails extensively, the cases of death registered as decline and consumption will on this account be in excess. Hence, we think it somewhat presumable that in those localities where, of the whole number of deaths registered, the proportion of consumption cases is great, the existence of much scrofula may be inferred. We do not propose this scheme as an undoubted test, but as one furnishing an *approximation* to the probable fact.

We take the several towns, analysed with respect to these matters, as they occur in the Report of the Registrar-general. Thus, in the exceedingly mixed population of the metropolis, out of 45,441 deaths, 7104 were in 1839 registered as from consumption, being in the proportion of 1 in $6\frac{1}{3}$; in the cotton districts of Manchester and Salford, 9223 deaths furnished 1454 cases of consumption, being in the same proportion as in the metropolis; in commercial Liverpool, all but exempt from manufactures of any kind, 9181 deaths gave 1762 instances of consumption, or 1 in $5\frac{1}{3}$; in the case of Leeds, the seat of the woollen manufacture, a total of 4388 yielded 804 of consumption, or 1 in 5½; and Birmingham, where there are no cotton mills, 3639 deaths included 668 cases of consumption, or 1 in every 5½. So it would appear that Manchester, with its factories, exhibits, in its fatal cases of disease, a somewhat moderate proportion of those here presumed to be of a scrofulous character. Just then to the extent that the test we have here applied may be considered to possess any value, it tends to disprove the notion regarding the extraordinary prevalence of scrofula in the districts of the cotton manufacture.

In closing the present article, we think ourselves entitled from all that is gone before to conclude that no peculiar evils to health and life attach, necessarily, to manufacturing pursuits. That the position of the labouring classes, as a whole, is comparatively prejudicial in these respects, we believe to be pretty well made out; and this, more particularly in the case of such as inhabit the ill-conditioned localities in our large towns; and in so far as factories, and other corresponding places of labour, interfere with the right conditions of health, they of course lead to the production of disease and to the shortening of life. We think however that, upon a review of the facts and circumstances considered in the preceding pages, the evils afflicting the working classes in this point of view will be considered to appertain to their *domestic* rather than to their *industrial* relations; and yet, badly as we think of the hygienic state of many of our cities, we cannot go the lengths of Sir A. Carlisle in his parliamentary evidence, in concluding that, because he could not find a person of the fourth generation by both the father and mother's side in the city of London, therefore our towns would undergo depopulation in a comparatively brief space of time, if not refreshed by accessions from the country. When the migratory character of our town populations is considered, we do not think Sir A. Carlisle's fact very extraordinary; and, assuredly, it warrants no such conclusion as the one which he seems to have deduced. Great evils undoubtedly follow in the train of advancing civilization; but, in the phraseology with which we set out, they must be regarded, not as something inseparable from such a state of things, but as the result of that imperfection of humanity, which, in no instance, can anticipate with a view to provide for every possible consequence of social changes, allowing man to become enlightened, not in all things at once, but only progressively. In admitting most fully the evils of our present state of society, we cannot too much reprobate the folly of those who would fasten upon isolated points, and expend their humanity and energy in decrying special departments of industry, rather than with a more general philanthropy, and with a sounder judgment, in probing the real source of the attendant evil, so as to ascertain how its removal can be effected without the infliction of greater evils upon the community at large. When from party-spirit, or from excited imagination, or from eccentric modes of looking at particular subjects, statements are hazarded to the effect that, as before quoted, "in English factories everything which is valuable in childhood is sacrificed to an inferior advantage in childhood. You purchase your advantage at the price of infanticide; the profit thus gained is death to the child;" or, when a depopulation of cities in fifty years is proclaimed to be the natural consequence of their existing ills; the investigation of the real question is prejudiced; and men, revolting from the exaggeration, too readily shut out the prospect of the real evil, and deny its existence. It is much better to regard the entire subject from a more general point of view, and to discuss the sanatory condition of our labouring population as a whole. In this way, all irritating topics may be avoided, and an effective effort be calculated upon with a view to provide the appropriate remedies for the removeable ills that do actually exist.

---

